# Study on Multi-Parameter Physical Processes and Flashover Threshold of Silicone Rubber Plate During AC Discharge in Salt Fog

**DOI:** 10.3390/mi16111241

**Published:** 2025-10-31

**Authors:** Xiaoxiang Wu, Yanpeng Hao, Haixin Wu, Jikai Bi, Zijian Wu, Lei Huang

**Affiliations:** 1 School of Electric Power Engineering, South China University of Technology, Guangzhou 510640, China; 2 School of Electrical and Information Engineering, Zhengzhou University, Zhengzhou 450001, China

**Keywords:** silicone rubber plate, AC salt fog discharge, visible light images, infrared thermal images, fiber Bragg grating, interface temperature

## Abstract

External insulation of coastal power grids transmitting offshore wind power faces significant threats from salt fog flashovers. Current arc monitoring and early warning technologies for flashover are severely inadequate. Research on salt fog discharge processes and determining the threshold at the flashover brink for transmission equipment external insulation is crucial for ensuring the safe operation of coastal grids delivering offshore wind power. Fiber Bragg Grating (FBG), with its advantages of compact size, excellent insulation, and fast response, enables effective discharge monitoring and identification of the critical flashover state on external insulation surfaces. In this study, FBGs were embedded at the interfaces of typical external insulation specimens, including silicone rubber plates and epoxy resin plates, to conduct contaminated AC salt fog discharge tests. Synchronized measurements of visible light images, infrared thermal images, and FBG interface temperature were conducted to investigate the discharge physical processes on silicone rubber insulating surfaces and the flashover threshold based on FBG temperature rise rate. The results indicate that discharge process can be divided into three phases: arc initiation, extension, and flashover based on the characteristics of arc visible light images. By comparing arc locations in infrared and visible light images with the corresponding FBG interface temperature rise, the arc phase criterion of FBG interface temperature rise rate and position were proposed. Furthermore, through multiple experiments, it has been found that flashover occurs when both interface temperatures reached above 4.6 × 10^−2^ °C/s. This study provides a novel research methodology for physical process of external insulation discharge and flashover warning in coastal salt fog environments.

## 1. Introduction

Offshore wind power, characterized by high wind energy density, stable wind speed, and large power generation capacity per unit, represents a significant development direction in the field of renewable energy generation [1]. Islands, coastal areas, and onshore grid-connection projects for offshore wind power [2] are often exposed to unique salt fog environments [3]. Such environments exhibit high humidity and elevated salt content in sea breezes and fog. Sea salt particles constitute primary source of salt fog. Under atmospheric influences, saline droplets are carried into the air and dispersed inland, undergoing continuous splitting, recombination, and evaporation, eventually forming salt fog [4]. The increased conductivity of salt fog droplets leads to contamination accumulation on insulators and reduction in electrical strength [5]. Statistical reports on insulator flashover accidents [6,7] indicate that surface contamination is the main cause of such failures. In humid salt fog environments, the adhesion of various types of particulate matter can exacerbate flashover phenomena, thereby increasing maintenance costs [8,9]. Pollution flashover under salt fog conditions frequently results in large-scale and prolonged power outages, posing a serious threat to the secure operation of power grids.

The discharge process on insulating surfaces is accompanied by the generation of various characteristic signals, including electrical, optical, acoustic, and thermal phenomena. Scholars have conducted extensive research on these signals and proposed multiple methods for investigating discharge processes, such as leakage current measurement [10,11], ultrasonic detection [12,13], infrared imaging [14,15], ultraviolet imaging [16,17], and visible light imaging [18,19].

Jia et al. [20] used photon count, luminous area, and number of light spots to evaluate discharge intensity on insulators and achieved flashover early warning by assessing the discharge state. Li et al. [21] recorded the entire process of AC arc propagation along contaminated insulator surfaces with a high-speed camera, and elucidated the physical characteristics of AC pollution flashover arcs. Liu et al. [22] utilized ultraviolet imaging to monitor the operational status of insulating surfaces. Maadjoudj et al. [23] captured visible light images of arc discharges on plate samples using cameras to characterize the presence of arcs on insulation surfaces. Yuan et al. [24] proposed an intelligent diagnostic method for surface discharge based on visible light imaging and machine learning.

The AC discharge process on insulating surfaces under salt fog conditions involves not only electrical but also thermal phenomena [25]. Liang et al. [26] used infrared thermography to capture changes in temperature distribution during dry-band formation on the surface of an insulating plate within a fog chamber. Hao et al. [27,28,29] embedded FBGs at the interface between the housing and the core rod of composite insulators to systematically investigate interfacial temperature characteristics under the influence of partial arcs. Furthermore, by conducting discharge experiments on silicone rubber and epoxy resin specimens with embedded FBG sensors, Bi et al. [30] proposed a method based on interfacial temperature monitoring to track droplet deformation and predict flashover during discharge process. Wu et al. [31] proposed a dual judgment method for flashover warning of fiber optic composite insulators.

The studies above have investigated discharge process on insulating surfaces from various perspectives, including leakage current, visible light imaging, infrared temperature, and ultraviolet emission, representing electrical, optical, and thermal aspects, respectively, and have achieved notable results. However, pollution flashover discharge is a complex process involving multiple physical and chemical phenomena such as electrical, optical, and thermal interactions [32]. The existing studies have predominantly focused on single-parameter analyses, without adequately accounting for the synergistic effects of multiple parameters throughout the phases of external insulation pollution flashover.

Currently, the primary methods for classifying the AC pollution discharge process on insulation surface are as follows: Li et al. [21] categorized the AC discharge process into arc initiation, development, and flashover phases based on the extension speed of arc length peaks. Jiang et al. [33] proposed that the pollution discharge process on composite insulators lacks clearly distinguishable phases of discharge development. Wu et al. [19] classified the AC salt fog discharge process on composite insulators into two distinct phases: partial arc development phase and critical flashover phase. The arc in development phase exhibits intermittent extinction and re-ignition phenomena, while the critical flashover phase is characterized by continuous arc burning.

The existing methods for staging AC contamination discharge rely predominantly on the characteristics of arc visible light images, which lack objective quantitative criteria and result in ambiguous staging boundaries. Therefore, there is a critical need to introduce multi-parameter fusion criteria to achieve a clearer and more objective division of the discharge stages for contaminated silicone rubber plates under AC salt fog conditions.

This study embedded FBGs at the silicone rubber-epoxy resin composite interface to conduct AC salt fog discharge monitoring tests. The experiment involved synchronous measurement of test voltage, visible light images of the discharge, infrared thermography, and the central wavelength of the grating. By analyzing the arc location, surface temperature, and interfacial temperature characteristics during the discharge process, this research enables an FBG-based investigation into the physical mechanisms of discharge and threshold of temperature rise rate of flashover on insulating surfaces.

## 2. Materials and Methods

### 2.1. Detecting System

**Figure 1:**
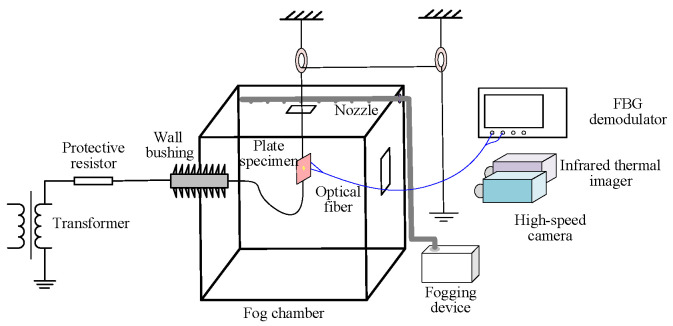
AC salt fog discharge test system.

A salt fog An AC salt fog discharge test system was established to collect electrical, thermal, and optical data during the discharge process on a contaminated silicone rubber plate, as illustrated in Figure 1. The test voltage was supplied by a single-phase transformer with a capacity of 50 kV/250 kVA [34].

The fog chamber was constructed with six transparent resin glasses, measuring 1.5 m × 1.5 m × 2 m (Length × Width × Height). The power supply cable on the left side was connected to the plate sample via a wall bushing. The right side was equipped with an observation window and an inlet for the salt fog pipeline. The fog generation device was placed outside the chamber. Salt fog was introduced into the chamber through the inlet pipe and distributed evenly via small holes arranged horizontally along the pipe.

A high-speed camera (Model: FASTCAM Mini AX100, Manufacturer: Tushuo Technology Co., Ltd., Shenzhen, China), was set up at 1 m away from the specimen, which was configured with a shooting speed of 4000 frames per second (FPS), shutter speed of 1/50,000 s, and maximum effective resolution of 892 × 766 pixels.

An infrared thermal imager (Model: TP96, Manufacturer: Hikvision Digital Technology Co., Ltd., Hangzhou, China), was used to measure the surface temperature of the insulator. This device features a measurement range of −20 to 650 °C, a resolution of 640 × 512 pixels, a spectral range of 7.5 to 14 μm, a frame rate of 50 FPS, and a temperature accuracy of ±2 °C.

Two customized optical fiber with a total length of approximately 3 m was used in this study to ensure that the measuring equipment could be located in a safe zone during high-voltage experiments. Each fiber was equipped with a single Bragg grating.

The interfacial temperature of the specimen was monitored by a FBG interrogator (Model: JEME-iFBG-S08, Manufacturer: Jiance Intelligent Technology Co., Ltd., Shenzhen, China). This device operates within a wavelength range of 1510 to 1590 nm, with a demodulation accuracy of 1 pm, a wavelength sensitivity of 0.1 pm, and a scanning frequency of 10 Hz.

### 2.2. Specimen and Method

**Figure 2:**
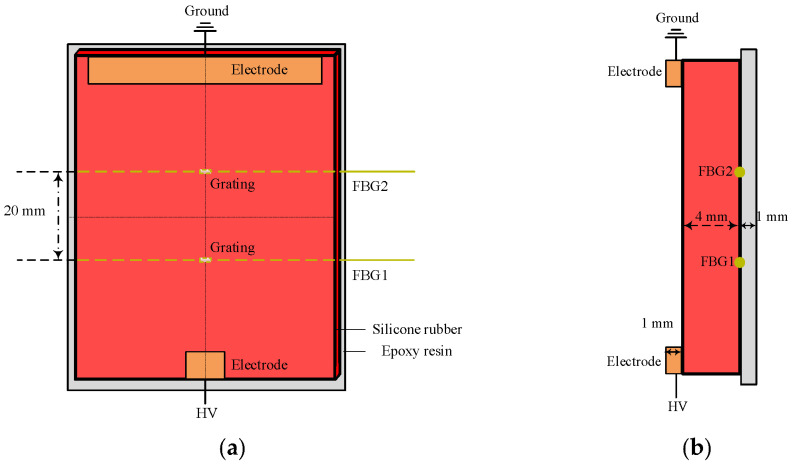
Grating distribution at the interface of plate specimen: (**a**) front view, (**b**) side view.

The specimen used for salt fog discharge experiment is shown in Figure 2. It consists of a silicone rubber plate and an epoxy resin plate, simulating the silicone rubber housing and the epoxy resin core rod of a composite insulator, respectively. The silicone rubber plate had a thickness of 4 mm. Two optical fibers inscribed with Bragg gratings were embedded at the interface between the silicone rubber and epoxy resin plates, with a spacing of 20 mm between the fibers. The two gratings were designated as FBG1 and FBG2.

Prior to the experiment, sodium chloride and kaolin were weighed according to specified pollution levels of 0.1 mg/cm^2^ for Equivalent Salt Deposit Density (ESDD) and 1.0 mg/cm^2^ for Non-Soluble Deposit Density (NSDD). The contaminants were mixed with water to form a paste-like suspension, which was then uniformly applied to the surface of the specimen to simulate external insulation contamination. The contaminated specimen was vertically suspended inside the fog chamber. The salt fog solution used during the test had a conductivity of 3 mS/cm, and its pH was maintained in the range of 6.5 to 7.2. The sedimentation rate of the salt fog was controlled between 1.0 and 2.0 mL/h per 80 cm^2^ of collection area. The diameter of the atomized droplets fell within the range of 1 to 5 μm. A timer was started at the beginning of the test to synchronously record visible light images of the arc, infrared thermography, and the central wavelength shifts in FBGs throughout various phases of the surface discharge.

### 2.3. FBG Temperature Measurement Principle and Calibration Experiment

**Figure 3:**
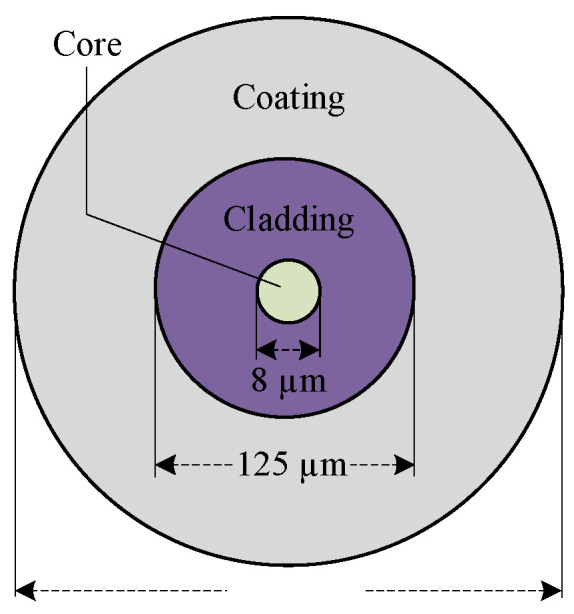
Schematic diagram of the optical fiber cross-section.

Optical fibers typically feature a coaxial cylindrical structure. From the inside out, the layers consist of the core, the cladding, and the coating [35], with the cross-sectional structure is illustrated in Figure 3. The core is composed of quartz fibers, while the cladding is primarily made of glass. The coating generally consists of synthetic polymer materials such as acrylic resin, polyurethane, or silicone resin. The core and cladding form the essential part of the optical fiber: the core serves as the primary pathway for light wave transmission, and the cladding ensures stable propagation by confining the light. When light enters the fiber, it undergoes total internal reflection at the interface between the core and cladding, propagating along the fiber axis. The region of the core that acts as the main propagation channel is referred to as the core mode. The primary function of the coating is to protect the fiber from environmental erosion and mechanical damage.

The FBG is an optical fiber segment exhibiting periodic modulation of the core refractive index. This periodic refractive index distribution enables the wavelength-selective reflection and transmission of light in the grating. As a reflective grating, its initial central wavelength is given by Equation (1) [36]:

(1) $$\lambda =2n_{eff}\cdot \Lambda_{T}$$

In this equation, $n_{eff}$ represents the effective refractive index of the fiber grating, which is a constant typically within the range of 1.446 to 1.485, with the specific value provided by the FBG manufacturer’s datasheet, and Λ_T_ denotes the grating period.

When the ambient temperature changes, the FBG experiences a wavelength drift due to the thermal expansion effect of the optical fiber, the thermo-optic effect that alters the refractive index, and the elasto-optic effect induced by thermal stress [37].

(2) $$\Delta \lambda =2\Delta n_{eff}\Lambda +2n_{eff}\Lambda \Delta \alpha$$

In the equation, *α* represents the thermal expansion coefficient. The variation in refractive index under the influence of temperature can be expressed as follows:

(3) $$\Delta n_{eff}=\frac{\partial n_{eff}}{\partial T}\Delta T+{\left(\Delta n_{eff}\right)}_{ep}+\frac{\partial n_{eff}}{\partial \alpha }\Delta \alpha$$

In the equations, *∂* $n_{eff}$*/∂T* represents the change in the refractive index of the fiber grating caused by the thermo-optic effect, generally denoted by *ξ* and referred to as the thermo-optic coefficient; ${\left(\Delta n_{eff}\right)}_{ep}$ corresponds to the elasto-optic effect induced by thermal expansion; *∂* $n_{eff}$*/∂α* indicates the waveguide effect resulting from the change in optical fiber diameter after thermal expansion. The complete expression for the relationship between temperature and wavelength in a fiber grating is thus given by:

(4) $$\frac{\Delta \lambda }{\lambda }=\left\{\frac{1}{n_{eff}}\left[\xi -\frac{n_{\mathrm{eff}}^{3}}{2}\left(P_{11}+P_{12}\right)\alpha +k_{wg}\alpha \right]+\alpha \right\}\Delta T$$

In the equation, k_wg_ represents the wavelength drift coefficient induced by the waveguide effect. As can be seen from the above equation, once the optical fiber material is determined, the wavelength sensitivity coefficient of the grating is typically a constant, which ensures a favorable linear wavelength output when using the FBG for temperature measurement. For fused silica optical fiber, its refractive index temperature coefficient *ξ* = 0.68 × 10^−5^/°C, and its linear thermal expansion coefficient α = 5.5 × 10^−7^/°C.

In summary, when the influence of external factors is not considered, the grating temperature sensitivity is primarily determined by the thermo-optic coefficient of the material, while the effects of the elasto-optic and waveguide contributions on the wavelength drift of the fiber grating are negligible. Therefore, Equation (4) can be simplified as:

(5) $$\frac{\Delta \lambda }{\lambda }=k\cdot \Delta T$$

The temperature coefficient of the FBG, *k*, fluctuates within 0.1%. In summary, a linear relationship exists between the shift in the grating’s central wavelength and the variation in temperature. To demonstrate the effectiveness of FBG in monitoring interface temperature changes, the temperature calibration of the FBGs embedded at the specimen interface was conducted in a temperature-controlled chamber. The chamber temperature was sequentially set to 0 °C, 5 °C, 10 °C, 15 °C, 30 °C, 35 °C, and 40 °C. After maintaining the specimen at each target temperature for 8 h, central wavelength of the grating was recorded. The average value within a 1 min period was taken as the central wavelength at the corresponding temperature.

### 2.4. Applied Voltage

**Figure 4:**
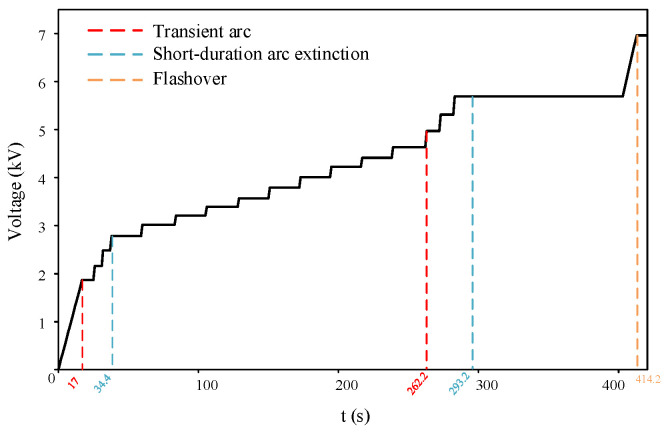
Voltage of AC discharge test on the specimen.

The typical voltage ramp-up curve for the AC salt fog discharge test on the silicone rubber plate is shown in Figure 4. At the beginning of the test, the voltage across the plate increased steadily until the initial arc appeared and extinguished rapidly. The voltage was then held constant, and data were recorded before resuming the voltage increase. This procedure was repeated iteratively until the arc propagated along the surface and eventually led to flashover.

## 3. Results and Analysis

### 3.1. Fiber Grating Temperature Calibration Results

**Figure 5:**
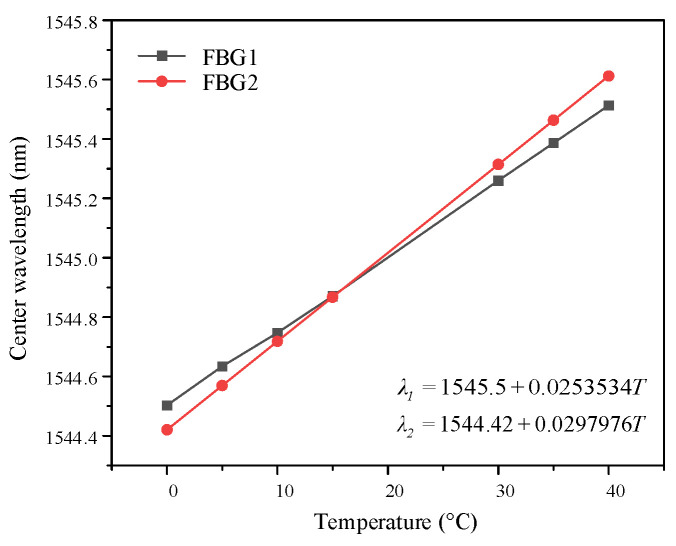
Linear fitting results of interface FBG temperature.

The temperature calibration results for the embedded FBGs at the specimen interface are shown in Figure 5. The temperature coefficient (*k_T_*) and the liner correlation coefficient of determination (*R*^2^) can be obtained by linearly fitting the central wavelength and temperature of each grating, as summarized in Table 1.

The *R*^2^ values of the linear fitting curves for both gratings exceeded 0.99, indicating a strong linear dependence between the central wavelength and temperature. Here, *λ*_1_ and *λ*_2_ represent the central wavelengths of the two gratings, both in units of nanometer (nm), and *T* denotes interface temperature in degrees Celsius (°C). The central wavelength shift in each grating exhibits a linear relationship with variation in interfacial temperature, as shown in the form Equation (6).

(6) $$\Delta \lambda =k_{T}\cdot \Delta T$$

where *k_T_* denotes the temperature coefficient of the grating in units of pm/°C. The temperature coefficients of FBG1 and FBG2 are 25.353 pm/°C and 29.798 pm/°C, respectively. Although variations in the temperature coefficients exist due to factors such as the embedding process of gratings and their interaction with surrounding materials, the calibrated coefficients remain effective for detecting interface temperature.

### 3.2. Evolution of Visible Image Morphology

**Figure 6:**
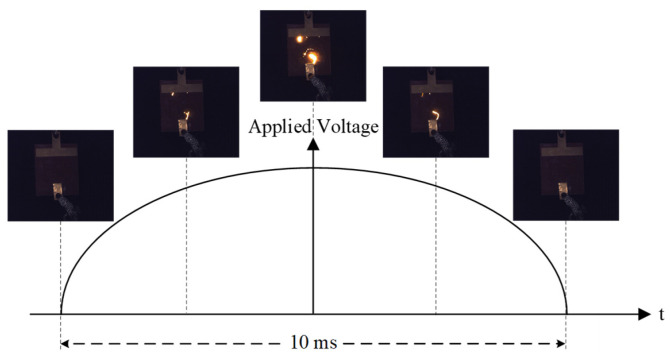
Periodic variation in the partial arc within a half cycle.

Under power-frequency voltage at 50 Hz, the arc exhibits a periodic “arc ignition-extinction” variation with a period of twice the power frequency. The arc ignites near the voltage peak and distinctly extinguishes around the voltage zero-crossing. The typical periodic behavior of the partial arc during this process is illustrated in Figure 6.

The AC salt fog discharge process can be categorized into three distinct phases: arc initiation, extension, and flashover.

**Figure 7:**
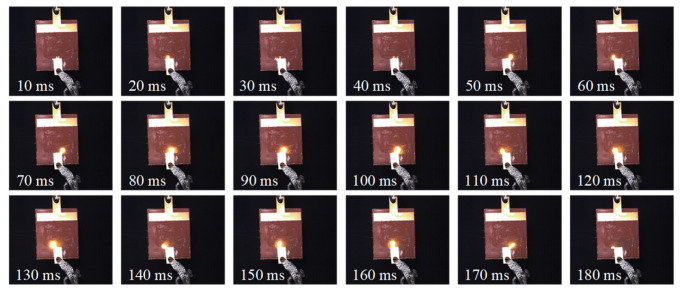
Evolution process of arc initiation phase (17 s).

(1) Arc initiation phase:

As shown in Figure 7, the arc initially appeared near the HV side. During this phase, arc activities were confined to the region close to the HV electrode. The moment when the arc first emerges is defined as the beginning of the initiation phase.

**Figure 8:**
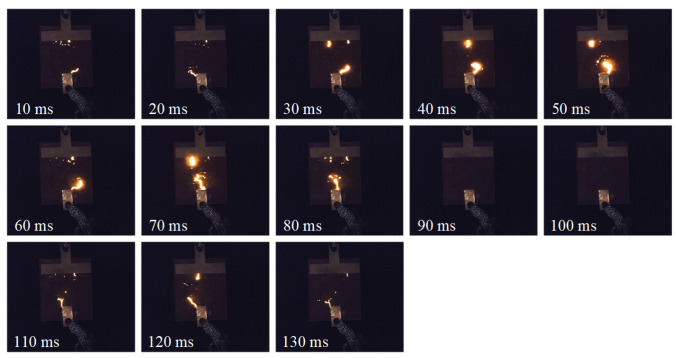
Evolution process of arc extension phase (262 s).

(2) Arc extension phase:

As shown in Figure 8, the arc propagated along the insulating surface, and discharges were observed near the grounded side for the first time in this phase. The moment when the arc initially occurs at the grounded side is defined as the start of the extension phase. Experiments have found that significant local arcs can be observed at most peak times of power frequency voltage. However, in these 13 consecutive discharge cycles, no discharge occurred when the voltage reached its peak at the 9th and 10th times. The lack of continuity indicates that there is a certain degree of randomness in the AC salt fog discharge on the surface of silicone rubber.

Under AC salt fog conditions, the contaminant layer on the silicone rubber plate surface is a dynamic and unstable system. Electrolysis, evaporation, ion migration, and residual effects from previous discharge cycles cause random, non-uniform distribution of layer thickness and conductivity at the microscopic scale. This dynamic inhomogeneity leads to localized distortion of the surface electric field, making discharge initiation and development highly dependent on instantaneous, stochastic local conditions. Even when the macroscopic voltage reaches relatively high levels, discharge in specific cycles may fail to occur if the electric field strength in the momentarily weakest region of the contaminant layer remains below the breakdown threshold of the air (or air-electrolyte mixture). Furthermore, results from multiple repeat tests under identical experimental conditions confirm that the phenomena observed in this study are not coincidental. Although the discharge generally follows the overall pattern of periodically enhanced “extinction-reignition”, its precise occurrence in each cycle and the duration of cycles exhibit significant randomness. This further demonstrates that the stochastic distribution of local surface states is the fundamental cause of the inherent uncertainty in this phenomenon.

**Figure 9:**
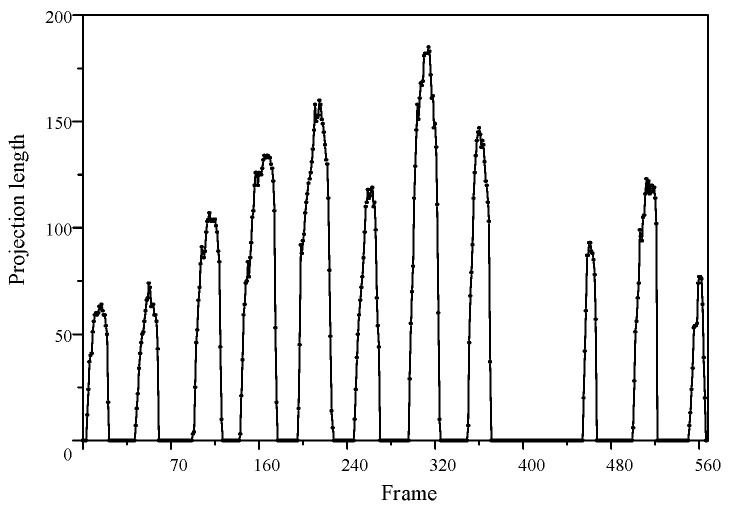
Temporal evolution of the arc vertical projection length.

As quantitatively demonstrated in Figure 9, the transition from the initiation to the development stage is marked by a sustained increase in the arc’s vertical projection length. The increase in the arc projection length metric provides measurable evidence of the arc extending from both electrodes toward the center, ultimately forming a complete bridging path.

**Figure 10:**
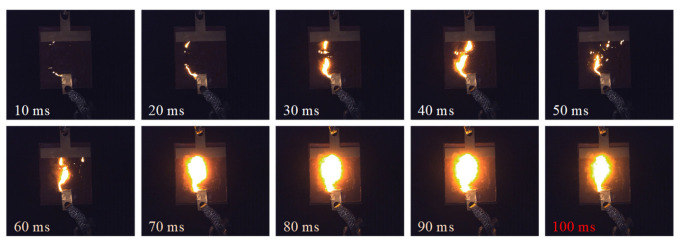
Evolution process of flashover phase (414 s).

(3) Flashover phase:

As shown in Figure 10, at 414.2 s into the test, the arc on the surface of the silicone rubber plate specimen began to intensify dramatically, with the discharge process lasting approximately 100 ms. During this period, a highly conductive discharge channel gradually formed between the two electrodes. Within the first 50 ms of the discharge development, the arc continued to extinguish at the zero-crossing point of the power frequency voltage. However, starting from approximately 60 ms into the discharge, the arc behavior underwent a significant transition: it persisted through the zero-crossing point and cyclically intensified along the carbonized or ionized discharge channel until final breakdown occurred, triggering a system protection trip.

### 3.3. Evolution of Infrared

**Figure 11:**
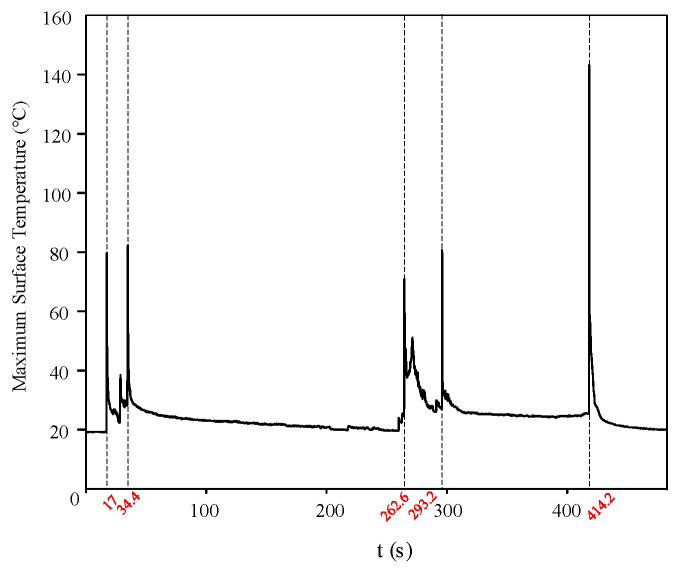
Maximum Surface Temperature of the Silicone Rubber Plate Sample under AC Salt Fog conditions.

Figure 11 presents the variation in the maximum surface temperature during the discharge process of the silicone rubber plate sample under AC salt fog conditions. A comprehensive analysis of the three typical phases of the discharge process is provided below.

**Figure 12:**
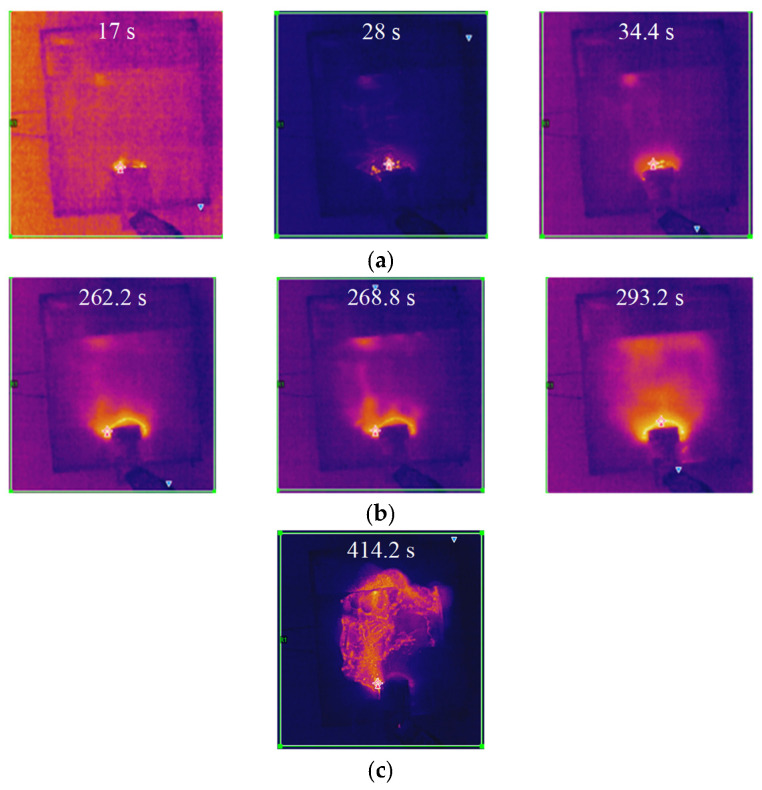
Evolution of infrared thermal imaging across discharge phases: (**a**) Arc initiation phase; (**b**) Arc extension phase; (**c**) Flashover phase.

(1) Arc initiation phase:

The test proceeded to the 17 s when a partial arc initially appeared in the dry zone near the high-voltage side electrode on the sample surface. During the period from 17 to 34.4 s, three distinct discharge processes were observed via infrared imaging, with the corresponding changes in surface temperature distribution shown in Figure 12a. During the arc initiation phase, although no visible arc was observed on the grounded side, the infrared thermal images indicated continuous heat accumulation in this area, suggesting that the discharge process was gradually transitioning into the arc development phase.

(2) Arc extension phase:

At 262 s, the arc on the specimen surface began to propagate from both electrodes toward the central region. During the period from 262 to 293.2 s, three distinct discharge processes were similarly detected by infrared imaging, with the corresponding variations in surface temperature distribution illustrated in Figure 12b. The infrared thermal images revealed that heat gradually extended from both electrodes toward the middle of the specimen during the discharge process, accompanied by the progressive formation of discharge channels, indicating that the discharge was advancing toward the final stage of bridging the positive and negative electrodes.

(3) Flashover phase:

The specimen experienced flashover at 414.2 s, with the corresponding infrared thermal image shown in Figure 12c. Under the salt fog contamination conditions, the electrical conductivity of the electrolyte solution on the silicone rubber surface increased, leading to elevated local current density under the electric field and significant Joule heating effects. As energy accumulated and thermal ionization progressed, these high-temperature regions bridged the discharge channel between the electrodes during the flashover process. This channel carried the majority of the current and induced intense gas ionization in the air, resulting in the observed floating arc phenomenon.

To quantitatively characterize the surface thermal behavior during localized discharge events, a quantitative analysis was performed on the infrared thermography data. Since significant changes in the infrared thermography only occurred during discharge events on the plate surface, the analysis focused on seven key discharge instances. For each corresponding thermography, the area ratio of the high-temperature region was calculated using an adaptive threshold-based image segmentation algorithm. The obtained high-temperature area ratios and the corresponding surface maximum temperatures for each discharge moment are summarized in Table 2. The progression of the high-temperature zones reveals the development of the discharge process. The expansion and eventual connection of these zones indicate the formation of a conductive discharge channel, which serves as a precursor to an impending flashover.

### 3.4. Evolution Behavior and Criteria of FBG Interface Temperature

**Figure 13:**
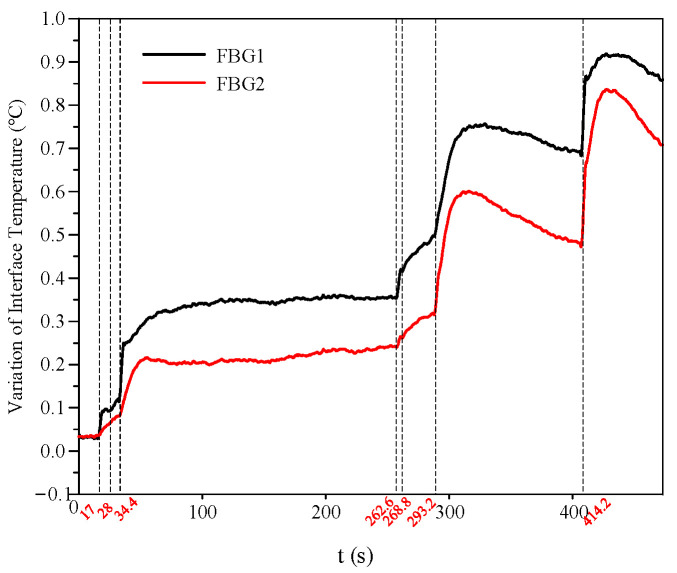
Variation in interface temperature during the discharge process of the silicone rubber plate specimen under AC salt fog conditions.

Under salt fog conditions, the variation in interface temperature during the AC discharge process of the silicone rubber plate specimen is shown in Figure 13. Throughout the entire discharge process, the interface temperature rise at FBG1 remained consistently higher than that at FBG2. Upon the occurrence of partial arcs, the interface temperatures at both FBG locations increased significantly. The following section provides a comprehensive analysis of the three typical phases of the discharge process and proposes criteria based on the location and rate of interface temperature rise in each phase. The interface temperature rise rate *β* discussed in this paper is defined as follows: taking the current moment as the starting point, it is the maximum temperature rise rate within a 10 s time window; if the actual temperature rise duration is less than 10 s, the average temperature rise rate from that moment until the temperature reaches its maximum value is taken.

(1) Arc initiation phase:

During the arc initiation phase, the interface temperature rise at FBG1 remained consistently higher than that at FBG2, as FBG1 was located closer to the arc position. At the 28 s mark, the arc on the specimen surface was relatively weak, resulting in a correspondingly small interface temperature rise at that moment. The values of interface temperature change and temperature rise rate for each FBG in this phase are summarized in Table 3.

(2) Arc extension phase:

During the arc extension phase, scattered arcs began to appear near the grounded electrode and between dry zones in the contamination layer. As a result, the interface temperature rise rate at FBG2 was significantly higher than during the initial arc phase and surpassed that of FBG1 at 293.2 s. At 268.8 s, the arc on the specimen surface was relatively weak, corresponding to a comparatively small interface temperature rise at that moment. The interface temperature change and temperature rise rate for each FBG in this phase are summarized in Table 4.

(3) Flashover phase:

During the flashover phase, the partial arc rapidly developed and bridged the positive and negative electrodes, forming a complete discharge channel. The interface temperature change and temperature rise rate for the specimen during this phase are summarized in Table 5.

In this study, FBG-h and FBG-g are defined as the FBGs installed on the high-voltage side and the grounded side, respectively. The criterion for arc initiation can be summarized as follows: the interface temperature at FBG-h rises rapidly, while that at FBG-g increases only marginally. The criterion for arc development is characterized by a rapid temperature rise at both FBG-h and FBG-g, with the rate of temperature increase at FBG-g beginning to exceed that at FBG-h. The criterion for flashover is identified when the interface temperatures at both FBG-h and FBG-g rise abruptly and reach their maximum values recorded during the entire discharge process. Based on repeated experiments under identical conditions, we have verified that the critical flashover interfacial temperature rise rate for composite insulators with different structures, but a silicone rubber thickness of 3.2 mm is around 4.6 × 10^−2^ °C/s, as shown in Table 6. This finding demonstrates strong consistency in the temperature-based flashover criterion across varied structural configurations under AC salt fog discharge conditions.

The critical temperature rise rate *βc* identified in this work was obtained under specific experimental conditions. While it serves as a strong characteristic signal preceding flashover in silicone rubber, its universality and quantitative relationship with parameters such as thickness and interface characteristics require further verification and refinement through more systematic experiments in the future.

### 3.5. Comparative Analysis of Monitoring Techniques

To better evaluate the applicability of different monitoring techniques, a systematic comparison among FBG, high-speed camera, and infrared thermal imager for flashover detection is presented in Table 7.

The comparison reveals that FBG technology holds an inherent advantage in response speed, achieving microsecond-level detection, which is significantly faster than infrared detection limited by thermal inertia and also surpasses high-speed photography constrained by exposure time and frame rate. Most critically, FBG sensors are characterized by intrinsic safety and complete immunity to electromagnetic interference (EMI). This is a decisive, irreplaceable advantage for field monitoring in high-voltage electrical equipment environments where strong electromagnetic fields are present. Furthermore, its high-temperature resistance, explosion-proof nature, and capability to form distributed measurement networks make it exceptionally suitable for long-term, stable online monitoring in complex and harsh industrial environments. Although high-speed cameras provide intuitive process recording and infrared thermal imagers can detect temperature distribution, FBG, with its unique comprehensive advantages, demonstrates superior reliability and irreplaceability in this specific application scenario.

## 4. Conclusions

This study proposes a multi-parameter approach for analyzing AC salt fog discharge by integrating FBG sensing, visible and infrared images. Through the synergistic interpretation of multi-source data, the discharge process was observed to exhibit an “extinction–reignition” periodicity at twice the power frequency, alongside a degree of stochastic behavior, as captured by visible light imaging. Concurrently, infrared thermal imaging revealed the spatiotemporal evolution of surface heating, showing a progressive thermal diffusion from the high-voltage side to the grounded side along the contamination layer. The study elucidates the intrinsic relationship between interface temperature rise and the evolution of discharge phases. The spatial correspondence of arc locations observed in both visible and infrared images validated the effectiveness of FBG temperature measurements. Furthermore, quantitative identification criteria were established: the initiation phase is dominated by temperature rise on the high-voltage side; the development phase is characterized by the grounding-side temperature rise rate surpassing that of the high-voltage side; and the flashover phase exhibits an abrupt temperature surge on both sides, with a critical threshold of 4.6 × 10^−2^ °C/s identified for early warning. By advancing the role of FBG from a mere temperature sensor to a core tool for diagnosing discharge dynamics, this work provides a novel theoretical basis and a practical technical pathway for real-time, quantitative diagnosis and early warning of surface discharge on composite insulators.

## Figures and Tables

**Table 1:** Temperature coefficient and liner correlation coefficient of determination of FBGs.

Gratings	*k_T_* (pm/°C)	*R* ^2^
FBG1	25.353	0.999
FBG2	29.798	0.993

**Table 2:** Quantified high temperature zone area ratio and maximum surface temperature during discharge events.

Time (s)	Proportion of High Temperature Zone (%)	Maximum Temperature (°C)
17	4.34	80.1
28	4.86	38.1
34.4	5.59	82.6
262.2	7.41	71
268.8	8.04	51.3
293.2	17.64	81
414.2	14.87	144.9

**Table 3:** FBG interface temperature rise rate during the arc initiation phase.

Time (s)	Gratings	*β* (°C/s)
17	FBG1	1.86 × 10^−2^
	FBG2	4.76 × 10^−3^
28	FBG1	4.58 × 10^−3^
	FBG2	1.99 × 10^−3^
34.4	FBG1	4.15 × 10^−2^
	FBG2	9.8 × 10^−3^

**Table 4:** FBG interface temperature rise rate during the arc extension phase.

Time (s)	Gratings	*β* (°C/s)
262.2	FBG1	2.06 × 10^−2^
	FBG2	9.13 × 10^−3^
268.8	FBG1	3.1 × 10^−3^
	FBG2	2.53 × 10^−3^
293.2	FBG1	1.48 × 10^−2^
	FBG2	2.54 × 10^−2^

**Table 5:** FBG interface temperature rise rate during flashover phase.

Time (s)	Gratings	*β* (°C/s)
414.2	FBG1	4.6 × 10^−2^
	FBG2	4.87 × 10^−2^

**Table 6:** Statistics of critical temperature rise rate β for silicone rubber samples.

Sample Type	Thickness (mm)	*β* (×10^−2^ °C/s)					Mean ± Standard Deviation
		Test 1	Test 2	Test 3	Test 4	Test 5	
Plate	4.0	4.6	4.55	4.68	4.62	4.71	4.63 ± 0.06
Composite Insulator	3.2	4.88	4.79	4.95	4.91	4.85	4.88 ± 0.09

**Table 7:** Comparison of flashover detection techniques.

Technical Indicator	FBG	High-Speed Camera	Infrared Thermal Imager
Detection Principle	Reflected wavelength shift	Visible light imaging	Infrared radiation imaging
Response Speed	Extremely fast (μs level)	Fast (ms level)	Slow (s level)
Spatial Resolution	Point/Quasi-distributed	Very High	High
Anti-EMI Capability	Strong	Strong	Strong
Environmental Adaptability	High-temperature resistant, explosion-proof, corrosion resistant	Requires viewing window, high explosion-proof requirements	Requires viewing window, affected by ambient temperature
Distributed Measurement	Easily achievable	No (Single field of view)	No (Single field of view)
Primary Advantages	Intrinsic safety, strong anti-interference, fast response, long lifespan, suitable for long-term monitoring in harsh environments	Intuitive, can record physical process	Can detect temperature anomalies
Primary Limitations	Direct measurement is strain/temperature	High cost, large data volume, requires light/line-of-sight	Unable to detect non-thermal faults, Susceptible to environmental effects

## Data Availability

The raw data supporting the conclusions of this article will be made available by the authors on request.
